# Adverse Events During Pregnancy Associated With Entecavir and Adefovir: New Insights From a Real-World Analysis of Cases Reported to FDA Adverse Event Reporting System

**DOI:** 10.3389/fphar.2021.772768

**Published:** 2022-01-03

**Authors:** Renjun Yang, Nuoya Yin, Ying Zhao, Dandan Li, Xuanling Zhang, Xingang Li, Yang Zhang, Francesco Faiola

**Affiliations:** ^1^ State Key Laboratory of Environmental Chemistry and Ecotoxicology, Research Center for Eco-Environmental Sciences, Chinese Academy of Sciences, Beijing, China; ^2^ College of Resources and Environment, University of Chinese Academy of Sciences, Beijing, China; ^3^ Department of Pharmacy, Beijing Friendship Hospital, Capital Medical University, Beijing, China; ^4^ Beijing Key Laboratory of Molecular Pharmaceutics and New Drug Delivery Systems, State Key Laboratory of Natural and Biomimetic Drugs, School of Pharmaceutical Sciences, Peking University, Beijing, China

**Keywords:** pregnancy, entecavir, adefovir, FAERS, disproportionality analysis, reporting odds ratio

## Abstract

**Background:** Due to the embryotoxicity found in animal studies and scarce clinical data in pregnant women, it is still controversial whether entecavir (ETV) and adefovir dipivoxil (ADV) are safe during human pregnancy. This is of paramount importance when counseling pregnant women with hepatitis B virus (HBV) on risks and benefits to their offspring.

**Objective:** To quantify the association between administration of ETV and ADV in pregnant women and occurrence of adverse events (AEs) during pregnancy (AEDP).

**Methods:** Pregnancy reports from the FDA Adverse Event Reporting System (FAERS) were used to perform a retrospective analysis of AEDP associated with ETV or ADV. Disproportionality analysis estimating the reporting odds ratio (ROR) was conducted to identify the risk signals. A signal was defined as ROR value >2, and lower limit of 95% confidence interval (CI)> 1.

**Results:** A total of 1,286,367 reports involving AEDP were submitted to FAERS by healthcare professionals. Of these, there were 547 cases reporting ETV and 242 cases reporting ADV as primary suspected drugs. We found a moderate or strong signal for increased risk of spontaneous abortion when comparing ETV with tenofovir disoproxil fumarate (TDF) and telbivudine (LdT), with RORs equal to 1.58 (95% CI, 1.09–2.30) and 2.13 (95% CI, 1.04–4.36), respectively. However, when the included reports were limited to indication containing HBV infection, no signals for increased AEDP were detected. Futhermore, a strong signal for increased risk of spontaneous abortion was identified in patients with HBV infection when comparing ETV or ADV with lamivudine (LAM), with RORs of 3.55 (95% CI, 1.54–8.18) and 2.85 (95% CI, 1.15–7.08), respectively.

**Conclusion:** We found a strong signal for increased risk of spontaneous abortion in patients with HBV infection taking ETV or ADV, in comparison with those prescribed with LAM. Moreover, no obvious signal association of human teratogenicity with exposure to ETV or ADV was identified in fetuses during pregnancy. Nevertheless, owing to the limitations of a spontaneous reporting database, which inevitably contains potential biases, there is a pressing need for well-designed comparative safety studies to validate these results in clinical practice.

## Introduction

Chronic infection with hepatitis B virus (HBV), an important global health problem, contributes to more than half of primary liver cancers worldwide (McGlynn et al., 2021). In the past decade, antiviral therapies for HBV have made great progress, and the benefits of treatment with nucleoside/nucleotide analogues (NAs) including lamivudine (LAM), adefovir dipivoxil (ADV), entecavir (ETV), telbivudine (LdT), and tenofovir disoproxil fumarate (TDF), are clear (Yuen et al., 2018). To date, no NAs are approved, in their drug labels, for administration during pregnancy. However, women in child-bearing age with HBV-related liver diseases may still need antiviral therapies, including during pregnancy, in order to reduce viremia and the risk of mother-to-child transmission (MTCT) (Terrault et al., 2021). Currently, LAM, LdT, and TDF are more commonly prescribed to pregnant women due to some evidence of their safety (Terrault et al., 2018; Sali et al., 2019; Funk et al., 2021). ETV and ADV are not recommended for pregnant women because of the embryotoxicity and congenital abnormalities found in animal studies, although the super-therapeutic dosages of ETV and ADV used in pregnant rat and rabbit animal models were almost 10 to 1000-fold higher than what is used in human beings (Giles et al., 2011). Nonetheless, the history of thalidomide emerging as a human teratogen serves as a lesson in drug development, giving us reasonable warnings that species differences exist in drug reactions or responses, and that animal reproductive toxicity studies are not always predictive of human response (Vargesson 2019).

It is still controversial whether the therapeutic dosage of ETV and ADV are unsafe in humans during pregnancy, due to sporadic clinical practice for pregnant women taking those drugs intentionally. Cases of pregnancy exposure to ETV or ADV caused by factors such as unintended pregnancies, are constantly emerging in real-world situations, evidenced by the increasing data in the Antiretroviral Pregnancy Registry (APR) and U.S. Food and Drug Administration Adverse Event Reporting System (FAERS) database (Antiretroviral Pregnancy Registry Steering Committee, 2021). Accordingly, information on the safety of ETV or ADV in pregnancy, and especially their risk on adverse events (AEs) during pregnancy (AEDP), are of paramount importance when counseling pregnant women with HBV about risks and benefits to their offspring.

The FAERS database, established for national post-market surveillance for drug safety, is a spontaneous reporting system for healthcare professionals, consumers, and drug manufacturers (Sakaeda et al., 2013). As FAERS case reports have been increasing annually, the database is largely used to identify novel drug-associated AEs not previously observed in clinical trials (Chen et al., 2021; Eugene et al., 2021; Ma et al., 2021). Moreover, data-mining of drug-related case reports from spontaneous reporting systems can provide us with a valuable source of information about the safety of specific drugs in real-life, especially for frail populations such as pregnant women (Tkachenko et al., 2019; Carnovale et al., 2020). By now, several methods have been used to evaluate the drug safety profile through mining the data from FAERS and other post-market spontaneous surveillance programs (Caldito et al., 2021; Xu et al., 2021). Of these, the disproportionality analysis estimating the reporting odds ratios (RORs) is a validated method to detect spontaneous signals, identifying the statistical association between a drug exposure and a specific AE (Caldito et al., 2021). ROR, calculated by the case/non-case method, represents the odds ratio of a particular AE to all the other AEs, for each drug (Rothman et al., 2004).

In the past decades, no pharmacovigilance studies were reported using the FAERS database specifically to address the potential risk of ETV and ADV, in pregnant women. Hence, we performed a retrospective observational study by data-mining of the FAERS database, and aimed at quantifying the association between the administration of the two drugs in pregnant women and the occurrence of AEDP.

## Methods

### Data Source

To estimate the safety of ETV and ADV during pregnancy, a retrospective analysis of the U.S. Food and Drug Administration’s reports from Q1/2004 to Q1/2021 was performed by using FAERS, a database designed for post-marketing safety surveillance for drugs (Sakaeda et al., 2013; Chen et al., 2021). For each report, the FAERS database contains information including suspected drugs (drug name, role codes, dose, and indication), AEs coded using terms in the Medical Dictionary for Regulatory Activities (MedDRA) terminology, patient demographics (gender, age, and weight), reporter occupation (physician, pharmacist, health-care professional, consumer, or manufacturer), patient outcomes, and therapy start and end dates.

### Data Extraction and Processing

Data were obtained from the FAERS public dashboard, in which each event was classified by reaction terms. Only cases involving AEDP (reaction terms coded within the category “pregnancy, puerperium and perinatal conditions”) were included, while cases pertaining to lactating women and male patients were excluded. Both generic names and brand names were used to identify the drugs in the database (Wishart et al., 2018). To reduce the potential risk of a misleading relationship between drugs and AEs, we only included reports entered by healthcare professionals. Duplicate records were removed, as previously described (Chen et al., 2021).

### Intervention and Comparisons

In this study, we conducted three comparisons. First, we compared ETV and ADV to all the other drugs. Second, we compared the two drugs to LAM, LdT, and TDF, respectively. Those three antiviral agents, supposed to have a low risk of AEDP (Terrault et al., 2018; Sali et al., 2019; Funk et al., 2021), were selected as reference drugs for relative comparison because they belonged to the same pharmacological class and were used for similar diseases. Third, we compared ETV and ADV to LAM, LdT, and TDF in HBV patients only, to decrease the potential risk of bias caused by different diseases, as LAM and TDF were also used in patients with human immunodeficiency virus (HIV)’s infection.

### Statistical Analysis

Cases were defined as reports where a drug induced a specific AE, while all other possible pairs were considered as non-cases. The Standardized MedDRA Queries associated with pregnancy-related topics were used to investigate the risk of particular clusters of AEs in our cases. The main AEDP investigated in our study were abortion, spontaneous abortion, preterm birth, low birth weight, stillbirth and foetal death, as well as foetal complications. As multiple drugs were sometimes reported for a unique report, to increase the confidence of the results, only the primary drug directly suspected of causing the AEs in each case report was considered in our study, while those listed as secondary suspected drugs, interacting or concomitant ones were excluded.

To assess the signals of disproportionate reporting for AEDP involving the drugs of interest, the analysis was conducted using ROR. The frequency of an AE of interest linked to our selected drugs was compared with that of reference drugs. In this study, ETV and ADV were our drugs of interest, while TDF, TdL, and LAM were considered as reference drugs. Sub-analysis was then performed, applying a filter to only include reports in which the “indication” for drug use was exclusively HBV infection. A signal of increased AE risk was defined as ROR value higher than 2, with the number of cases higher than 3, lower limit of 95% confidence interval (CI) greater than 1, and Chi square value higher than 4 (Sakaeda et al., 2013). ROR between one and two was considered as a moderate signal (Guo et al., 2021). An ROR value lower than one was deemed not to indicate a potential safety signal. Data tidying and statistical analysis were performed using R software (version 3.6.2) and Stata 12.0.

## Results

### Overall Events

During the study period (over 17 years, from Q1/2004 to Q1/2021), a total of 1,286,367 reports involving AEDP were submitted to FAERS by healthcare professionals. Of these, 547 cases concerned ETV and 242 involved ADV as primary suspected drugs (Table 1). For reference drugs, the number of cases listing TDF, LdT, and LAM as primary suspected drugs was 3750, 313, and 4364, respectively. As the level of completeness of reported information varied from case to case, we applied a filter to include reports with the “indication” of drug use exclusively for HBV infection, excluding reports with other indications (such as HIV), or without indication. As shown in Table 1, there were 305 and 225 reports related to ETV and ADV with HBV-related indication, respectively.

**TABLE 1:** Number of interest and reference drugs’ reports submitted for each adverse event in the United States Food and Drug Administration Adverse Event Reporting System (FAERS).

Adverse event	No. of reports with events				
	ETV	ADV	TDF	LdT	LAM
*All indications*					
Abortion	49	14	174	11	149
Spontaneous abortion	36	12	160	10	143
Preterm birth	26	2	302	13	208
Low birth weight	8	0	21	3	11
Stillbirth and foetal death	10	2	105	3	150
Foetal complications	47	23	382	28	385
Total	547	242	3750	313	4364
*HBV indication*					
Abortion	25	14	43	7	11
Spontaneous abortion	20	12	35	6	8
Preterm birth	13	2	25	10	6
Low birth weight	0	0	1	0	0
Stillbirth and foetal death	6	2	22	1	22
Foetal complications	6	18	76	5	96
Total	305	225	840	131	413

ETV, entecavir; ADV, adefovir dipivoxil; TDF, tenofovir disoproxil fumarate; LdT telbivudine; LAM, lamivudine.

### AEDP for ETV and ADV Compared to all the Other Drugs

**FIGURE 1:**
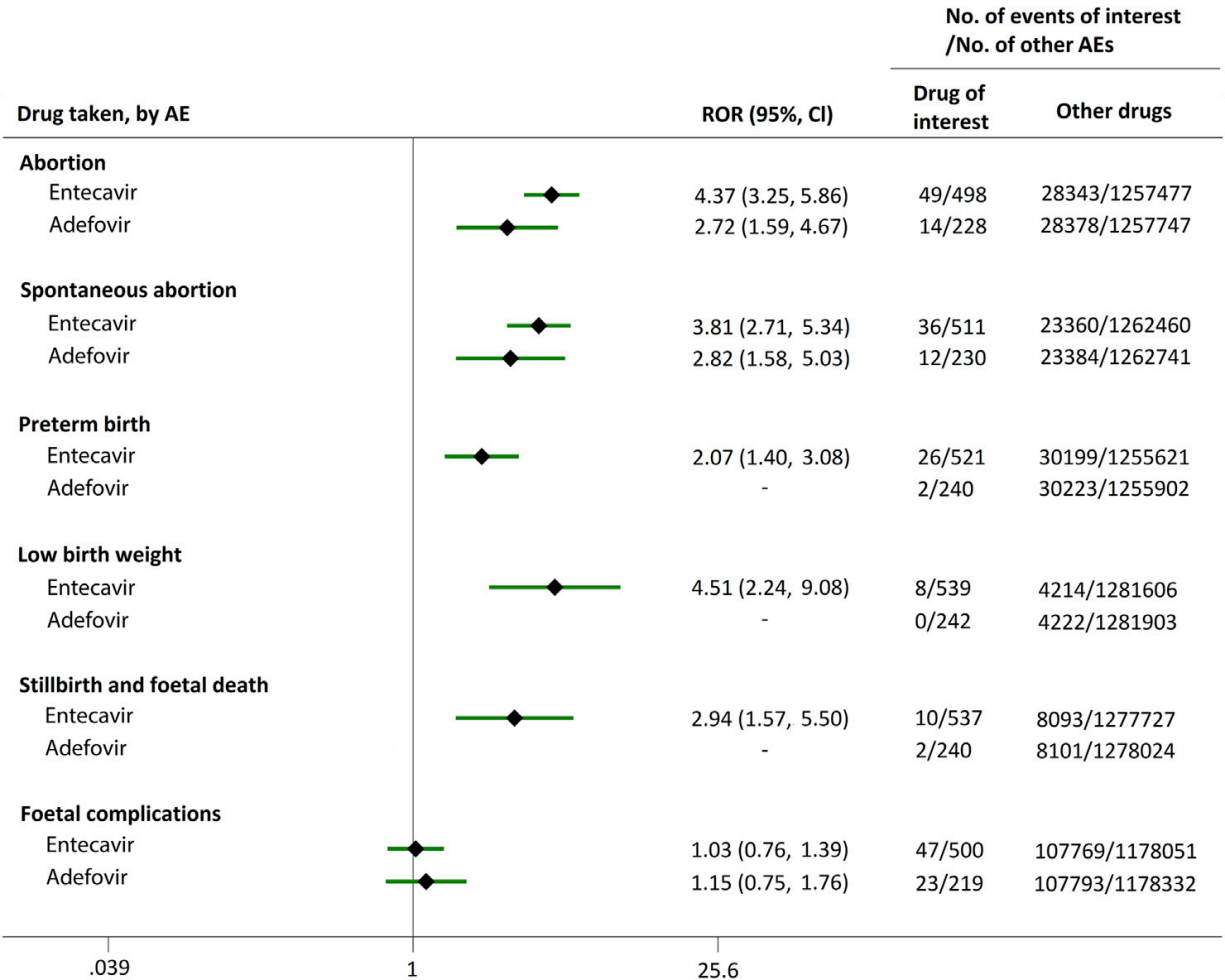
Reporting odds ratios (RORs) of adverse events during pregnancy (AEDP) for entecavir (ETV) or adefovir (ADV), compared to all the other drugs.

As mentioned in the method section, each AE was automatically classified in an AE category (abortion, spontaneous abortion, preterm birth, low birth weight, stillbirth and foetal death, and foetal complications) according to the preferred terms (PTs) in MedDRA. For instance, the AE category of abortion contained abortion early, abortion threatened, abortion induced, abortion spontaneous, habitual abortion, imminent abortion, etc. As shown in Figure 1, when comparing ETV or ADV with all the other drugs, there were strong signals for increased abortion risk in pregnant women, with ROR values of 4.37 (95% CI, 3.25–5.86) and 2.72 (95% CI, 1.59–4.67), respectively. Furthermore, reports of spontaneous abortion were substantially more common with ETV (ROR, 3.81; 95% CI, 2.71–5.34) and ADV (ROR, 2.82; 95% CI, 1.58–5.03) than with the other drugs. In addition, ETV was associated with higher proportion of AEs in preterm birth, low birth weight, and stillbirth and foetal death categories, as compared to the other drugs, with RORs of 2.07 (95% CI, 1.40–3.08), 4.51 (95% CI, 2.24–9.08), and 2.94 (95% CI, 1.57–5.50), respectively. Reports of foetal complications were similar between ETV and ADV, and all the other drugs, with RORs of 1.03 (95% CI, 0.76–1.39) and 1.15 (95% CI, 0.75–1.76), respectively, showing no signals for increased risks of foetal complications.

### AEDP for ETV, ADV, and Their Nucleoside/Nucleotide Analogues

**FIGURE 2:**
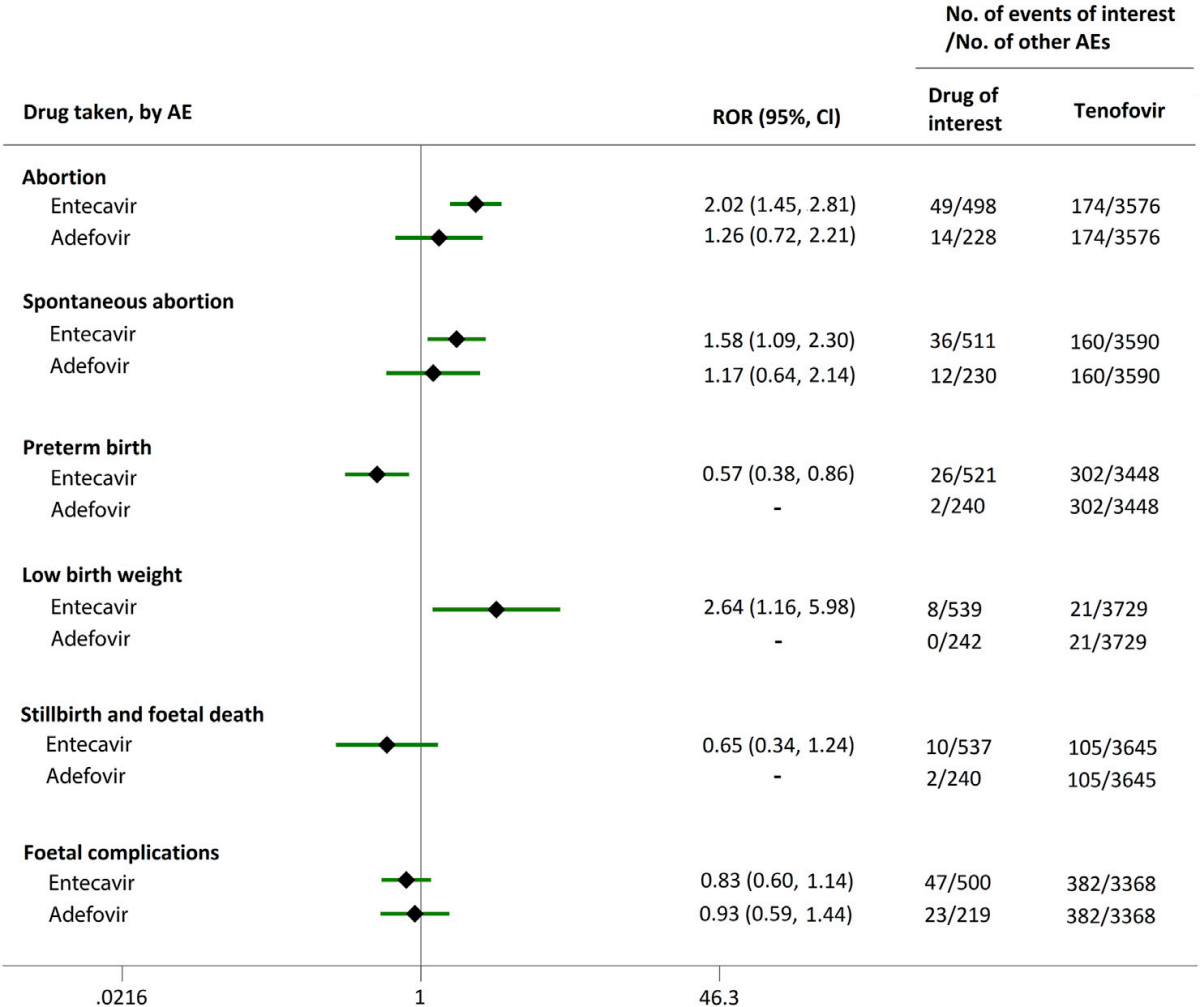
Reporting odds ratios (RORs) of adverse events during pregnancy (AEDP) for entecavir (ETV) or adefovir (ADV), compared to tenofovir (TDF).

**FIGURE 3:**
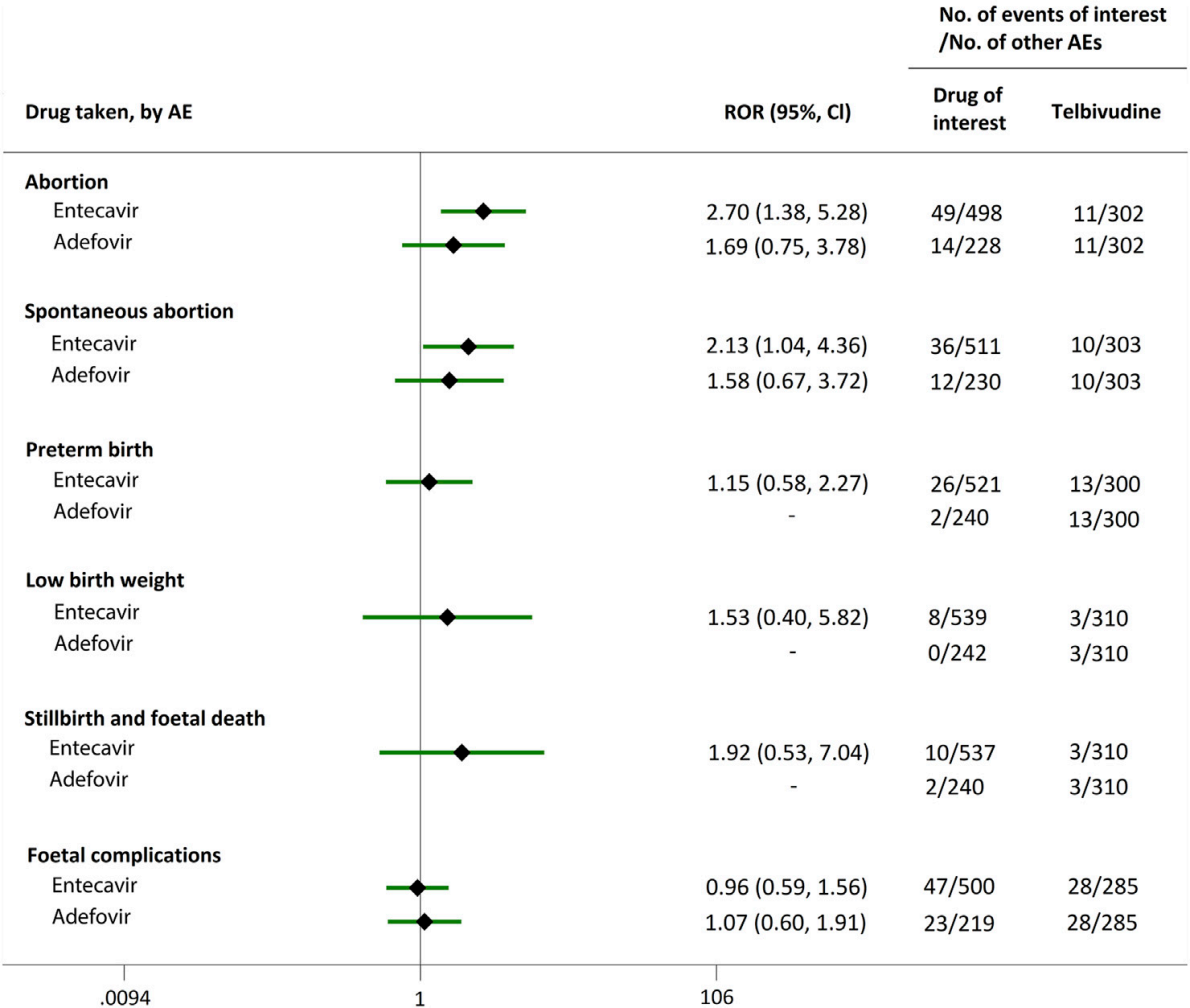
Reporting odds ratios (RORs) of adverse events during pregnancy (AEDP) for entecavir (ETV) or adefovir (ADV), compared to telbivudine (LdT).

**FIGURE 4:**
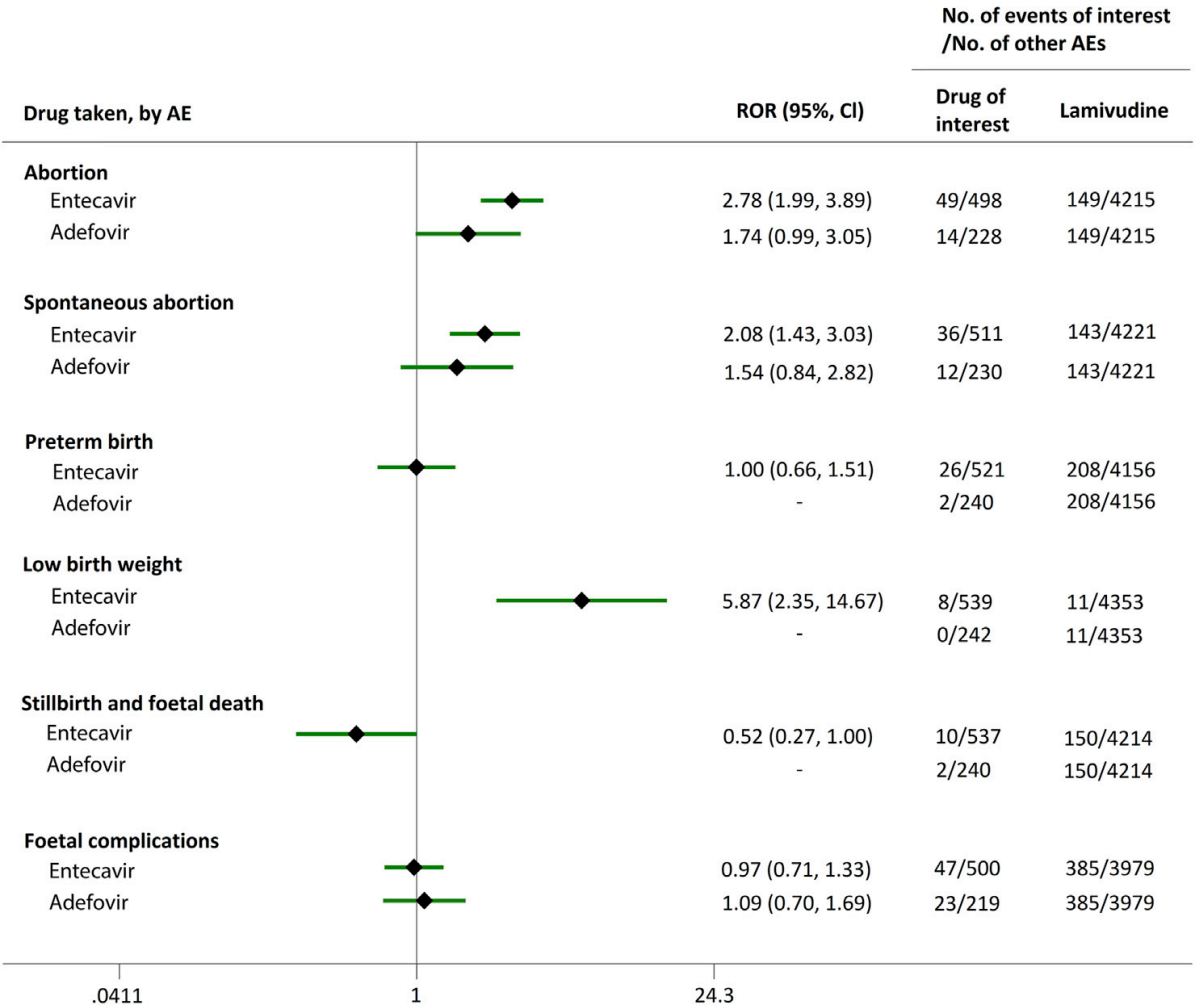
Reporting odds ratios (RORs) of adverse events during pregnancy (AEDP) for entecavir (ETV) or adefovir (ADV), compared to lamivudine (LAM).

Direct comparisons of the proportion of reports for AEDP were made between drugs of interest (ETV and ADV) and reference drugs (TDF, LdT, and LAM), which are all NAs used for antiviral therapies. As shown in Figure 2, when comparing ETV with TDF, the ROR values for abortion, spontaneous abortion, and low birth weight were 2.02 (95% CI, 1.45–2.81), 1.58 (95% CI, 1.09–2.30), and 2.64 (95% CI, 1.16–5.98), respectively, suggesting strong signals for increased abortion and low birth weight risks in pregnant women, in parallel with a moderate signal for increased spontaneous abortion risk. Meanwhile, the proportion of AEs in preterm birth, stillbirth and foetal death, and foetal complications were similar or lower, with RORs of 0.57 (95% CI, 0.38–0.86), 0.65 (95% CI, 0.34–1.24), and 0.83 (95% CI, 0.60–1.14), respectively (Figure 2). When the reference drug was set to LdT, strong signals of increased abortion and spontaneous abortion risks were detected for ETV, with ROR values of 2.70 (95% CI, 1.38–5.28), and 2.13 (95% CI, 1.04–4.36), respectively (no signals for other AEDP, Figure 3). When ETV was compared to LAM, the ROR values for abortion, spontaneous abortion, and low birth weight were 2.78 (95% CI, 1.99–3.89), 2.08 (95% CI, 1.43–3.03), and 5.87 (95% CI, 2.35–14.67), respectively, with no disproportionate reporting identified for preterm birth, stillbirth and foetal death, and foetal complications (Figure 4).

Futhermore, as shown in Figures 2–4, reporting rates of abortion, spontaneous abortion, and foetal complications were similar between ADV and the reference drugs TDF, LdT, and LAM, showing no signal of increased AEDP.

### AEDP in HBV Patients

**FIGURE 5:**
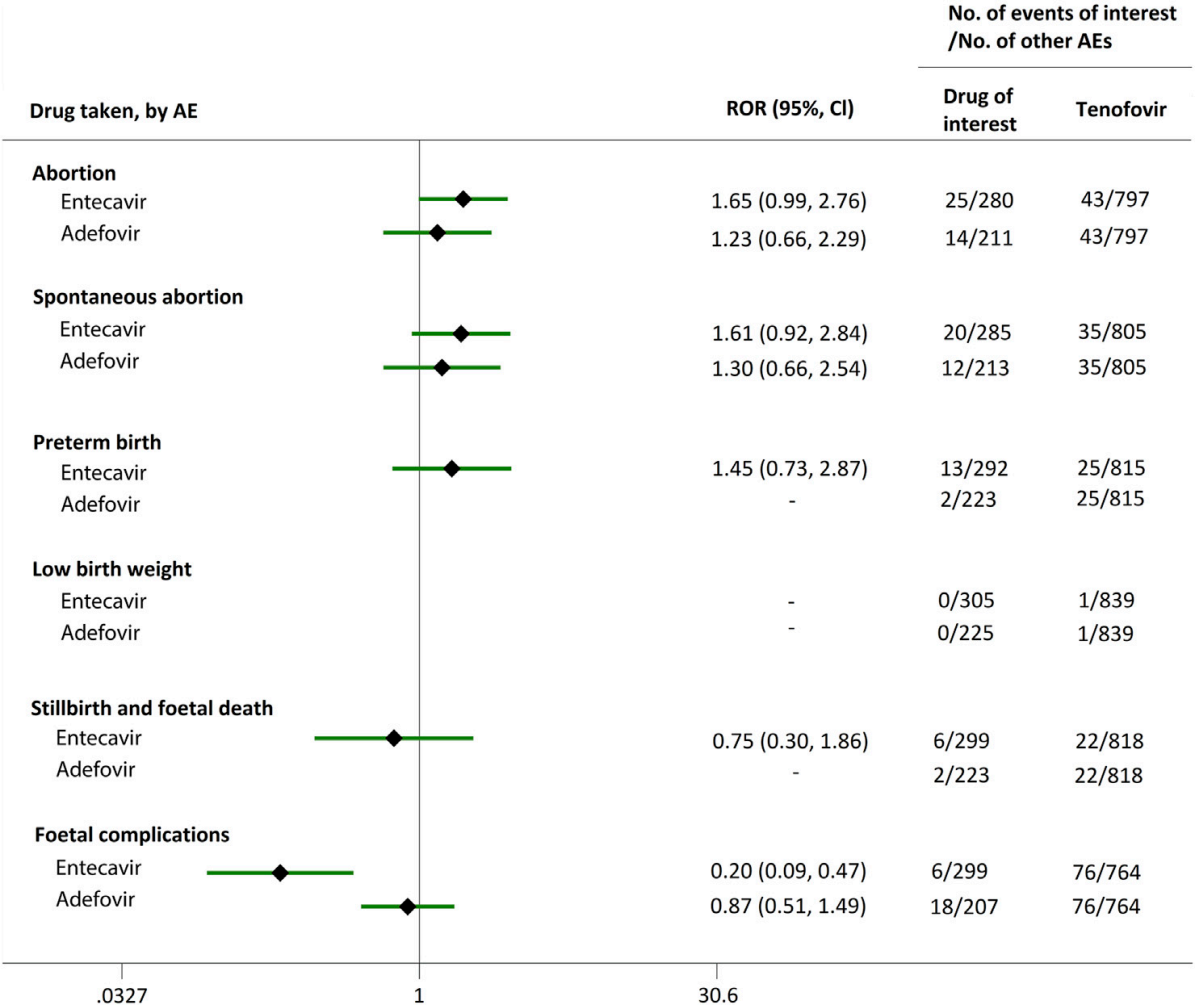
Reporting odds ratios (RORs) of adverse events during pregnancy (AEDP) for entecavir (ETV) or adefovir (ADV) in patients with HBV infection, compared to tenofovir (TDF).

**FIGURE 6:**
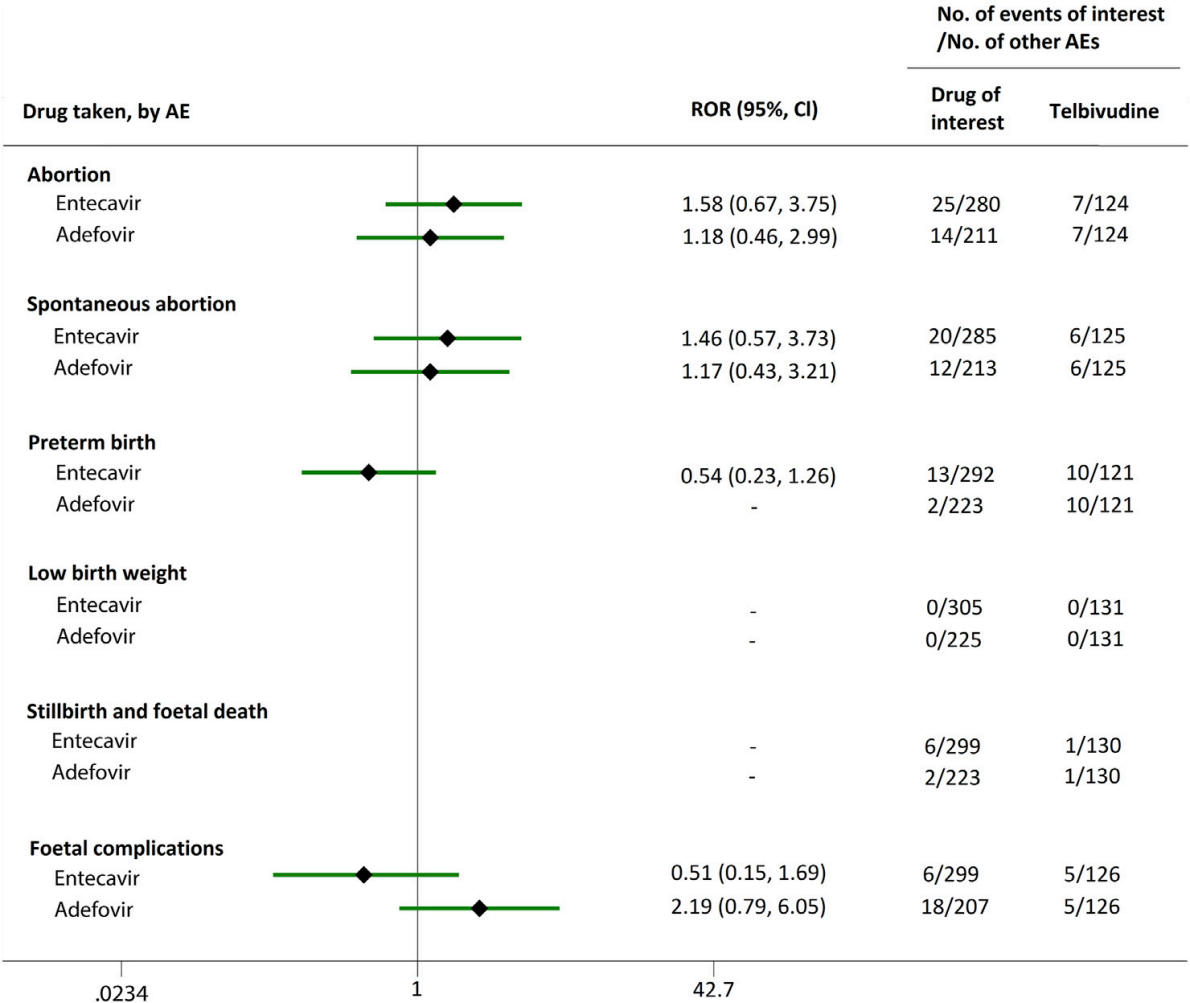
Reporting odds ratios (RORs) of adverse events during pregnancy (AEDP) for entecavir (ETV) or adefovir (ADV) in patients with HBV infection, compared to telbivudine (LdT).

**FIGURE 7:**
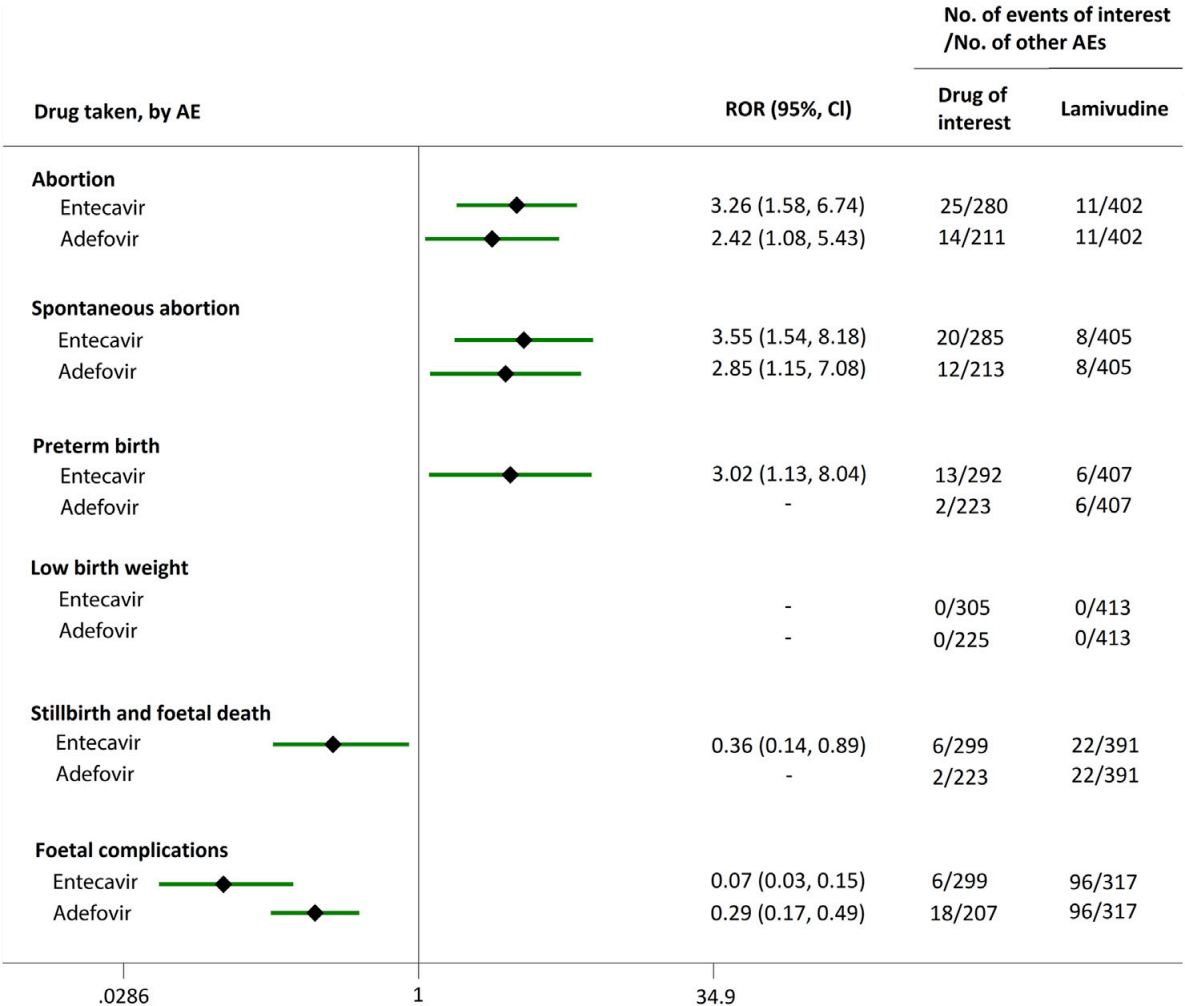
Reporting odds ratios (RORs) of adverse events during pregnancy (AEDP) for entecavir (ETV) or adefovir (ADV) in patients with HBV infection, compared to lamivudine (LAM).

We extended a sub-analysis for drugs of interest (ETV and ADV), in comparison with the reference drugs (TDF, LdT, and LAM), only in reports with the indication of HBV infection, to help control for confounding bias. According to their ROR and 95% CI values, in comparison with TDF (Figure 5) and LdT (Figure 6), ETV and ADV did not present any disproportionate reporting for all the PAE clusters. When the reference drug was set to LAM, the results shown in Figure 7 indicated that both ETV and ADV probably had a lower safety profile owing to higher reporting rates of abortion and spontaneous abortion, with RORs of 3.26 (95% CI, 1.58–6.47) and 3.55 (95% CI, 1.54–8.18), respectively, for ETV, and 2.42 (95% CI, 1.08–5.43) and 2.85 (95% CI, 1.15–7.08), respectively, for ADV. In addition, ETV was associated with higher proportion of preterm birth AEs, as compared to LAM, with an ROR of 3.02 (95% CI, 1.13–8.04).

## Discussion

In general, decisions about initiating drug therapy for HBV infection in pregnant women must be made carefully, considering the risk-benefit profile of the mother and fetus. Based on the limited available data about the safety of NAs, the American Association for the Study of Liver Disease (AASLD) supports the use of TDF, LdT, and LAM during pregnancy (Terrault et al., 2018). Currently, ETV and ADV are not recommended for pregnant women based primarily on preclinical animal studies as no relevent prospective clinical studies were carried out in humans. However, species and dosage differences make it difficult to determine whether the toxicity observed in pregnant animals after exposure to ETV or ADV could be translated to humans administered with the drug’s therapeutic dosage. To date, there are only limited cases available in the scientific literature regarding the safety of ETV or ADV in unintended pregnant women (Kakogawa et al., 2011; Gao et al., 2020; Kakiuchi et al., 2021). As far as we know, our study is the first disproportionality analysis utilizing real-world data from a large pharmacovigilance database to identify the association between the adiministration of ETV or ADV in pregnant women and the occurrence of AEDP. Taking everything into consideration, this study can provide worthwhile insights on the safety profile of ETV and ADV in pregnant women who were often excluded from clinical trials.

The FAERS database, one of the largest spontaneous reporting and publicly available databases for AEs, includes contributions by healthcare (physicians, pharmacists, and others) and non-healthcare (consumers, drug manufacturers, etc.) professionals (Sakaeda et al., 2013). The analysis of the FAERS database has been exploited in pharmacovigilance for drug safety assessments, not only to detect drug-AE associations unidentified in pre-market clinical trials, but also protect patients from potential harms (Chen et al., 2021; Eugene et al., 2021; Ma et al., 2021). It is worth noting that whether a drug is the causative agent of an AE occurring during its use, is something that can only be accurately judged through a rigorous causality assessment by healthcare professionals, while non-healthcare professional reports on their own appear to increase the risk of erroneous information (Andreaggi et al., 2020). Thus, only reports entered by healthcare professionals were included in our study.

The FDA previously rated ETV and ADV as pregnancy category C agents, although that category classification is no longer applied due to oversimplified risk-benefit profile of the mother and fetus. Partly as a result, very few cases of patients treated with ETV or ADV during pregnancy have been reported in the literature (Kakogawa et al., 2011; Gao et al., 2020; Kakiuchi et al., 2021). Limited reported cases may indicate that some women chose to continue administration of ETV or ADV for HBV treatment during pregnancy, with no evidence of increased incidence of birth defects in infants or higher risk of AEs for mothers (Kakogawa et al., 2011; Yu et al., 2011; Gao et al., 2020; Kakiuchi et al., 2021). Generally, small-scale clinical studies are not robust enough to address drug safety. In the real-world situation, a large proportion of pregnancies are unplanned, entailing unintentional prescription of drugs potentially unsafe for pregnant women (Sheffield et al., 2014). Therefore, information from spontaneous reporting systems could be very useful to guarantee the safety profile of drugs, and should help us broaden the knowledge of ETV and ADV administrations in pregnancy.

As shown in Table 1, in the FAERS database, ETV-associated AEDP were more than those of LdT, although ETV is not recommended for pregnant women while LdT is considered as a candidate to treat HBV infection during pregnancy (Terrault et al., 2018). This phenomenon could be explained by the fact that, as a first-line NA drug for the treatment of HBV infection (Brown et al., 2016), ETV was more frequently prescribed in clinical practice, and more women became accidently pregnant during antiviral therapy with ETV. Although ADV and LdT are not used very often in the U.S., considering our study period lasted over 17 years (from Q1/2004 to Q1/2021) and that the AEs were collected from different regions (Terrault et al., 2018; Chen et al., 2021), it is reasonable that there were reports involving ADV and LdT in FAERS. Table 1 showed that the number of cases listing ADV and LdT as primary suspected drugs was 242 and 313, respectively. Those were fewer than the cases concerning TDF and LAM, 3750 and 4364, respectively. Available data assessed from the Antiretroviral Pregnancy Registry (APR) show no difference in the overall rates of birth defects for TDF or LAM, compared with those rates in the general population (Antiretroviral Pregnancy Registry Steering Committee, 2021). Although APR data on fetal safety for LdT remain limited, systematic reviews demonstrated that TDF, LdT, and LAM were safe during pregnancy, with no increased risk of any infant or maternal adverse outcomes (Brown et al., 2016; Terrault et al., 2018; Terrault et al., 2018; Sali et al., 2019; Wu et al., 2020; Funk et al., 2021).

LdT, ADV and ETV are only approved, in their drug labels, for administration during HBV infection. There are clinical trials reporting the efficacy of ETV and ADV for the treatment of HIV (Miller et al., 2001; Sasadeusz et al., 2008). However, as shown in Supplementary Table S1, there were no HIV reported cases in FAERS for LdT and ADV, while 13 cases were reported for ETV with HIV indication. Since a significant number of cases had no indication and our study included cases over a 17-year period, we cannot rule out whether those patients were taking the drugs to treat hepatitis B or were participating in clinical trials for HIV. Hence, to decrease the potential risk of bias caused by different diseases, we applied a filter to include only reports with HBV indication, and exclude reports with HIV indication, or no indication.

The proportion of abortion in the ETV group was significantly higher than that of TDF and LdT. We speculate that the fear of ETV harmful effects on their offspring made some women choose to have an abortion, especially in unintentional pregnancies. Although a moderate or strong signal for increased risk of spontaneous abortion was identified for ETV, as compared to TDF and LdT (Figures 2, 3), such signal disappeared when only case reports with HBV infection indication were considered (Figures 5, 6). Furthermore, ETV did not present any disproportionate reporting for preterm birth, stillbirth and foetal death, and foetal complications in pregnancy with HBV infection, in comparison with TDF and LdT (Figures 5, 6). These results are in accordance with the ones obtained from a small-scale retrospective study conducted in China, which enrolled 20 pregnant women with chronic hepatitis B and treated with ETV or ADV in the first trimester of pregnancy (Gao et al., 2020). These findings may therefore help reduce patients’ obsession whether to consider abortion or not in the event of an unexpected pregnancy after taking ETV. Nevertheless, in comparison with LAM, ETV showed a strong signal for increased risk of spontaneous abortion, while no signals for increased risk of stillbirth and foetal death, as well as foetal complications, were detected. In addition, except for higher rates of abortion and spontaneous abortion in patients with HBV infection, when comparing ADV with LAM, there appeared not to be any association with any safety signals for abortion, spontaneous abortion, and foetal complications for ADV, relative to TDF or LdT, in line with a previous study with small sample size (Gao et al., 2020). On the whole, in comparison with the reference drugs (TDF, LdT, and LAM), we did not find any obvious signal association of human teratogenicity in fetuses during pregnancy after exposure to ETV or ADV.

NAs, including LAM, ADV, ETV, LdT, and TDF, are either purine or pyrimidine modified with various types of groups (heterocyclic or chain-like), resulting in different polarities and pharmacokinetic properties. For example, the elimination half-lives for ETV, LdT, TDF, and LAM are 129.9–148.9 h (Scott and Keating., 2009), 40 h (Keam. 2007), 14.4 h (Perry and Simpson., 2009), and 6.2 h (Jarvis and Faulds., 1999), respectively. One study suggested that placental barrier’s functions may be affected by hepatitis virus infections, evidenced by expression of placental drug transporters and consequent selection of antiviral drugs (Pfeifer et al., 2018). Pregnant women with hepatitis B infection may be more prone to drug cumulative toxicity owing to defective placental barrier. The long elimination half-time of ETV may impact fetal drug exposure. This fact may be one reason why ETV is not as safe as LdT, TDF or LAM during pregnancy.

Several strengths could be mentioned for this study. First, we provided a head-to-head assessment of pregnancy-related safety signals for NAs in one of the largest pharmacovigilance databases, considering it is almost impossible to conduct a prospective clinical study to evaluate the safety profile of NAs in the mother and fetus during pregnancy, due to ethical concerns. Second, unlike the previous case reports or small-scale retrospective studies mentioned above (Kakogawa et al., 2011; Gao et al., 2020; Kakiuchi et al., 2021), in which the included pregnant women treated with ETV or ADV were no more than 20, this study examined 789 women taking ETV or ADV during pregnancy, with a long study period of over 17 years (from Q1/2004 to Q1/2021), providing more convincing and relatively more valuable evidence for post-market surveillances of drug safety. Third, the FAERS database has the capability to capture some safety events such as abortion in pregnancy, which are not reported in the APR or missed in prospective studies (Antiretroviral Pregnancy Registry Steering Committee, 2021). Specially, the incidence of abortion was not reported in the APR, and “abortion early” was often missed in prospective studies since women were only included when they were pregnant rather than in their pre-conception period. Conversely, FAERS contains AEs related to abortion, such as abortion early, abortion threatened, abortion induced, abortion spontaneous, etc.

Of course, this study has also several limitations. First, owing to the spontaneous nature of FAERS reporting, there are some intrinsic limitations that make the data quality less than optimal, including missing, incorrect and misclassified information, overreporting, underreporting, and selective reporting, to name just a few (Sakaeda et al., 2013; Duggirala et al., 2016). Second, the lack of a denominator of medication users constrained our ability to estimate the true incidence of a specific AE. Third, in the FAERS database, it is not required to report the information of patients’ disease conditions like severity and seriousness, even though the existence of clinical heterogeneity is a potential confounder that should be considered. Take LAM as an example, it can be used to treat different diseases (like the ones caused by HBV and HIV). Patients taking LAM may have different health conditions, which are susceptible to be confounding variables in this study. Fourth, confounding by concomitant drugs (both other suspected and non-suspected drugs) and by co-morbid conditions could also have affected our results. However, it is almost impossible to evaluate and eliminate these uncontrolled factors in FAERS. Finally, the causal relationship between a drug-AE pair cannot be generated *via* disproportionality analysis alone. Also, in clinical practice, an increased ROR value is not always in parallel with a higher risk of AEs. Despite these limitations, FAERS database’s data-mining should be considered as exploratory to detect signals of unknown or unexpected AEs, rather than a validation or hypothesis testing of any causal relationship. The safety signal identified from FAERS data should warrant further investigations in clinical work.

## Conclusion

The present study, by using the FAERS pharmacovigilance database, suggests a moderate or strong signal for increased risk of spontaneous abortion when comparing ETV with TDF or LdT. However, when case reports were limited to indication containing HBV infection, no signal for increased AEDP was detected. Furthermore, a strong signal for increased risk of spontaneous abortion was identified in patients with HBV infection when comparing ETV or ADV with LAM. No obvious signal association of human teratogenicity with exposure to ETV or ADV was identified in fetuses during pregnancy. Finally, owing to the limitations of a spontaneous reporting database which inevitably contains potential biases, there is a pressing need for well-designed comparative safety studies to validate those results.

## Data Availability

The raw data supporting the conclusions of this article will be made available by the authors, without undue reservation.
