# Supplementary material for: Adverse Events During Pregnancy Associated With Entecavir and Adefovir: New Insights From a Real-World Analysis of Cases Reported to FDA Adverse Event Reporting System
Source: Front Pharmacol. 2022 Jan 3;12:772768. doi: 10.3389/fphar.2021.772768 (PMC8762051; doi:10.3389/fphar.2021.772768)
Supplement: Supplementary file 1 [file Table1.DOCX]

**Table S1** Number of interest and reference drugs’ reports submitted for each adverse event with HIV indication in the U.S. Food and Drug Administration Adverse Event Reporting System (FAERS)

| **Adverse event** | **No. of reports with events** | | | | |
| --- | --- | --- | --- | --- | --- |
|  | **ETV** | **ADV** | **TDF** | **LdT** | **LAM** |
| Abortion | 4 | 0 | 98 | 0 | 98 |
| Spontaneous abortion | 2 | 0 | 96 | 0 | 97 |
| Preterm birth | 0 | 0 | 160 | 0 | 112 |
| Low birth weight | 0 | 0 | 5 | 0 | 6 |
| Stillbirth and foetal death | 0 | 0 | 59 | 0 | 84 |
| Foetal complications | 0 | 0 | 193 | 0 | 191 |
| Total | 13 | 0 | 1799 | 0 | 2659 |

ETV entecavir, ADV adefovir dipivoxil, TDF tenofovir disoproxil fumarate, LdT telbivudine, LAM lamivudine
